# Endocrine Disruptors in the Workplace, Hair Spray, Folate Supplementation, and Risk of Hypospadias: Case–Control Study

**DOI:** 10.1289/ehp.11933

**Published:** 2008-11-20

**Authors:** Gillian Ormond, Mark J. Nieuwenhuijsen, Paul Nelson, Mireille B. Toledano, Nina Iszatt, Sara Geneletti, Paul Elliott

**Affiliations:** 1 Department of Epidemiology and Public Health, University College Cork, Cork, Ireland; 2 Department of Epidemiology and Public Health, Imperial College London, London, United Kingdom; 3 Center for Research in Environmental Epidemiology (CREAL), Municipal Institute of Medical Research (IMIM), CIBER Epidemiologia y Salud Pública (CIBERESP), Barcelona, Spain; 4 Independent Public Health Consultant, Phrisk Ltd, London, United Kingdom

**Keywords:** endocrine disruptors, hair spray, folate supplementation, hypospadias, occupation

## Abstract

**Background:**

Hypospadias is one of the most common urogenital congenital anomalies affecting baby boys. Prevalence estimates in Europe range from 4 to 24 per 10,000 births, depending on definition, with higher rates reported from the United States. Relatively little is known about potential risk factors, but a role for endocrine-disrupting chemicals (EDCs) has been proposed.

**Objective:**

Our goal was to elucidate the risk of hypospadias associated with occupational exposure of the mother to endocrine-disruptor chemicals, use of folate supplementation during pregnancy, and vegetarianism.

**Design:**

We designed a case–control study of 471 hypospadias cases referred to surgeons and 490 randomly selected birth controls, born 1 January 1997–30 September 1998 in southeast England. Telephone interviews of mothers elicited information on folate supplementation during pregnancy and vegetarianism. We used a job exposure matrix to classify occupational exposure.

**Results:**

In multiple logistic regression analysis, there were increased risks for self-reported occupational exposure to hair spray [exposed vs. nonexposed, odds ratio (OR) = 2.39; 95% confidence interval (CI), 1.40–4.17] and phthalate exposure obtained by a job exposure matrix (OR = 3.12; 95% CI, 1.04–11.46). There was a significantly reduced risk of hypospadias associated with of folate use during the first 3 months of pregnancy (OR = 0.64; 95% CI, 0.44–0.93). Vegetarianism was not associated with hypospadias risk.

**Conclusions:**

Excess risks of hypospadias associated with occupational exposures to phthalates and hair spray suggest that antiandrogenic EDCs may play a role in hypospadias. Folate supplementation in early pregnancy may be protective.

Hypospadias is one of the most common urogenital congenital anomalies affecting baby boys (Harris 1990). Prevalence estimates in Europe range from 4 to 24 per 10,000 births, depending on definition (Dolk et al. 2004), with higher rates reported from the United States (Paulozzi 1999). Since the 1960s, prevalence appears first to have increased, then stabilized, along with increasing trends among other male fertility-related disorders (Dolk et al. 2004; Toppari et al. 1996). Little is known about the etiology of hypospadias. A role for endocrine-disrupting chemicals (EDCs) has been proposed, specifically antiandrogens (Baskin et al. 2001). Animal studies suggest association of EDCs with various disorders related to male fertility (Sharpe et al. 2003), but data on humans are largely lacking (Committee on Toxicity 2006). Studies of occupational risk factors have been inconclusive (Aho et al. 2003; Irgens 2000; Kallen 1988; Kristensen et al. 1997; Pierik et al. 2004; Vrijheid et al. 2003; Weidner et al. 1998). Concerning diet and vitamin use, few data are available on effects of folic acid supplementation/antagonists (Brouwers et al. 2007; Czeizel 1996; Czeizel et al. 2001), and two studies reported excess risk related to vegetarianism (Akre et al. 2008; North and Golding 2000). We report here results of a large population-based case–control study of hypospadias in southeast England with primary aims of investigating risk of hypospadias and *a*) maternal occupational exposures, specifically to EDCs; *b*) folic acid supplementation during pregnancy; and *c*) vegetarianism.

## Data and Methods

**Figure 1:**
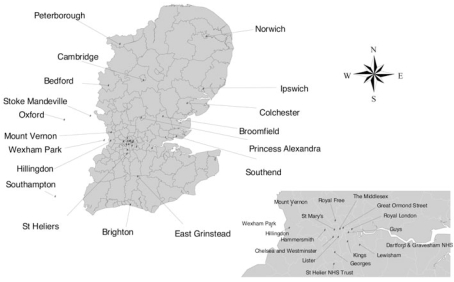
The study area and major National Health Service (NHS) hospitals operating on hypospadias in the southeast of England.

The study region included the health regions of North Thames, South Thames, and the Anglian part of Anglia and Oxford (Figure 1), comprising 120 London boroughs and local authority districts. Surgical centers in the study region and major surgical centers within 50 miles were visited. Hypospadias cases born in the study region over a 21-month period (1 January 1997–30 September 1998) were eligible if there was an abnormally positioned urethral orifice requiring surgery, with no major accompanying anomaly suggesting that it was part of a syndrome. Forty of 41 surgeons operating on hypospadias in the study region participated; 731 cases were identified from surgeons’ records and case notes. After initial contact by letter, up to two further invitations were sent. Of the 731 mothers, 610 replied, of whom 471 (77%; 64% of total eligible cases) agreed to participate.

Controls born in the study region during the same period as the cases were randomly selected from the birth registry by the Office for National Statistics (ONS; London, UK). Ethical approval was given by the West Midlands Multi-centre Research Ethics Committee, Birmingham, United Kingdom. Following guidelines from the ethics committee, ONS asked the health authorities (now primary care trusts) to contact the general practitioners (GPs) of the control children. The GPs were then asked to pass on the invitation to the mother of the control child; in turn, the mothers were asked to contact the study team. In total, 1,568 control mothers were selected, but letters were not sent out to 81 mothers. From the possible 1,487 invitees, there were 758 replies, of whom 490 (65%; 33% of total eligible controls) agreed to take part. We attempted to contact a sample (*n* = 200) of the 729 mothers who had not replied and found that 144 (72%) had not received an invitation, mainly because the GP had not passed on the information (73%); the family had moved without a forwarding address (9.5%); or for other reasons. The cases and control mothers who agreed to participate were interviewed by telephone between September 2000 and March 2003.

Case and control mothers were interviewed by telephone using a standard set of questions, with answers directly entered into a computer. The questionnaire included information on parental age, ethnicity, education, household income; family history of disease; pregnancy history; and maternal occupation, vegetarianism, folate supplements, smoking, alcohol use during pregnancy, and other questions related to diet history, vitamin use, demographics, and domestic and environmental exposures to chemicals.

Approval for the study was obtained from the Multi-centre Research Ethics Committee and local research ethics committees in the study area, and participating mothers gave written consent before taking part in the study.

### Occupational exposure to EDCs

To assess their occupational exposure to EDCs during the first 3 months of pregnancy, mothers were asked about their job title, department, company, their five main tasks, possible exposure to a list of 26 occupational substances (including hair spray, plastic fumes, cleaning agents such as disinfectants, solvents, paints and paint removers, printing ink, glue, heavy metal, welding and soldering fumes, anesthetics, cytostatics and antibiotics, and pesticides), and the hours per week that they were in contact with these exposures while at work.

We used a job exposure matrix that included 348 possible job titles (Van Tongeren et al. 2002) to classify job title, department, company, and main tasks into seven exposure categories assessed by a panel of occupational hygienists as to the likelihood of exposure to EDCs (Van Tongeren et al. 2002). We then dichotomized exposure into either exposed—including possible and probable exposure—or unexposed, blind to case or control status. The classes of EDCs were pesticides, polychlorinated organic compounds, phthalates, alkylphenolic compounds, biphenolic compounds, heavy metals, and other (Baskin et al. 2001; Vrijheid et al. 2003).

### Statistical methods

We assessed correlations between variables using Spearman’s and kappa statistics. Exposure prevalence for cases and controls and unadjusted odds ratios (ORs) and 95% confidence intervals (CIs) were calculated. Chi-square tests were used to test for statistical significance.

We performed multiple logistic regression in the statistical package R (version 2.2.0; R Development Core Team 2005). Variables were included in the multiple regression if they were statistically significant (*p* < 0.05) in the univariate model and improved fit of the multiple logistic regression model using Akaike Information Criteria (Akaike 1974). Positive family history of hypospadias and previous stillbirth were excluded from the multiple logistic regression models because among the control participants there were only one and two with the specific risk factor respectively. *p*-Values are uncorrected for multiple comparisons. Because of multicolinearity (kappa = 0.82), maternal occupational exposure to hair spray and phthalates were not entered together in the multiple logistic regression models. Income or level of education used to control for potential confounding by social class yielded similar findings; only models that included income are shown here. We also examined multiple logistic regression models that included maternal age.

## Results

Table 1 shows prevalence of various risk factors and potential confounders for hypospadias. Gestational age and birth weight were correlated (Spearman *r* = 0.41), as were income and maternal age (*r* = 0.33) and maternal smoking and environmental tobacco smoke (*r* = 0.31); no other correlations exceeded ± 0.30. The hypospadias cases were more likely to be born preterm and to have low birth weight (< 2,500 g) (Table 1). Compared with mothers of control children, mothers of hypospadias cases tended to have lower income (*p* = 0.001), higher prevalence of smoking (*p* = 0.02), and lower prevalence of folate supplement use during the first 3 months of pregnancy (*p* = 0.01) (i.e., the critical period for development of hypospadias) (Harris 1990). For these variables, unadjusted ORs for the association with hypospadias ranged from 1.44 (95% CI, 1.05–1.97) for smokers compared with nonsmokers, to 2.74 (95% CI, 1.55–4.92) for household income < £10,000 compared with > £50,000, whereas folate supplementation during the first 3 months of pregnancy was associated with significantly reduced risk (OR = 0.64; 95% CI, 0.46–0.89) (Table 1). There was no association with vegetarianism (*p* = 0.36). Positive family history of hypospadias and previous stillbirth were also significantly associated with risk of hypospadias (not shown).

Among occupational exposures (prevalence ≥ 5%), significantly increased risk was found for self-reported exposure to hair spray (exposed vs. nonexposed unadjusted OR = 2.30; 95% CI, 1.38–3.89) (Table 2). With the job exposure matrix, exposure prevalences were all < 5%; significant excess risk of hypospadias was found for boys of mothers exposed to phthalates (including hairdressers, beauty therapists/those working in beauty/hairdressing, research chemists, line operators, pharmaceutical operators, electrical assemblers, factory assistants) compared with those with no exposure to phthalates at work (unadjusted OR = 3.65; 95% CI, 1.19–11.20; 14 cases, 4 controls).

After adjustment for multiple potential confounders, the significant positive association with maternal occupational exposure to hair spray was slightly strengthened (exposed vs. unexposed OR = 2.39; 95% CI, 1.40–4.17) (Table 3). Hairdressers as a group had a nonsignificant increased risk (unadjusted OR = 2.73; 95% CI, 0.72–10.38; adjusted OR = 2.59; 95% CI, 0.70–12.32). Folate supplementation during the first 3 months of pregnancy remained inversely associated with risk of hypospadias, with a virtually unchanged risk estimate (OR = 0.64; 95% CI, 0.44–0.93). With phthalates included in the model instead of occupational exposure to hair spray, the risk estimates for folate supplementation and other factors remained essentially unchanged; occupational phthalates exposure was associated with 3-fold elevated risk (OR = 3.12, 95% CI, 1.04–11.46) (Table 3). Inclusion of maternal age did not materially alter the risk estimates for the other variables in the model (data not shown).

## Discussion

In this large case–control study, we found 2- to 3-fold increased risk of hypospadias among children of mothers exposed to hair spray and phthalates in the workplace during pregnancy and a 36% reduction in risk associated with folate supplementation during the first 3 months of pregnancy. These findings were robust to control for potential confounders. In contrast with two smaller studies (Akre et al. 2008; North and Golding 2000), we did not find an association between vegetarianism/veganism and hypospadias.

This is the first study to report a significant association between maternal occupational exposure to hair sprays, some of which may contain phthalates, and risk of hypospadias. A previous study reported that from 1980 to 1989, women hairdressers had slightly reduced risk of giving birth to a boy with hypospadias, whereas during 1992–1996, risk was significantly increased (OR = 1.50; 95% CI, 1.02–2.09) (Vrijheid et al. 2003). The risk was reduced after adjustment for parental social class (OR = 1.18; 95% CI, 0.80–1.64), suggesting possible confounding (Vrijheid et al. 2003). In contrast, our study showed that adjustment for household income (or maternal educational level) as a proxy for social class did not materially affect the risk estimates.

We are unaware of studies that have reported urinary phthalate metabolites in hairdressers or women applying hair sprays, so it is unclear to what extent their exposure may have been elevated at the time of the study. Phthalates, predominantly diethyl phthalate (DEP) and dibutyl phthalate (DBP), were present in many cosmetics including deodorants, fragrances, and nail and hair products (Hubinger and Havery 2006; Koo and Lee 2004). However, since 2005, certain phthalates including DBP have been prohibited for use in cosmetic products in Europe. The phthalates or their metabolites, for example, monoethyl phthalate and mono-*n*-butyl phthalate, are associated with androgen-lowering activities and abnormal Leydig cell function and have been linked to a decrease in anogenital distance in male infants (Main et al. 2006; Swan et al. 2005); androgen lowering is associated with reproductive tract malformations including hypospadias (Lottrup et al. 2006; Mylchreest et al. 2000, 2002). Inhalation contributes significantly to the uptake of these phthalates (Adibi et al. 2003), which may explain some species differences for DEP between human and animal studies, where oral administration has mainly been used (Lottrup et al. 2006; Mylchreest et al. 2000, 2002). A number of other substances included in hair sprays may have toxic effects if inhaled. They include polyvinyl alcohol, polyvinylpyrolidone, hydrofluoro-carbon, and propylene glycol, although for these substances the concern is acute effects on the cardiorespiratory system, skin, and eyes rather than on the reproductive system (U.S. National Library of Medicine 2008).

This is also the first study to show a protective effect of folate supplementation on risk of hypospadias. A recent Dutch case–control study found no association of maternal folic acid supplements and hypospadias (Brouwers et al. 2007), nor was there an effect in a trial of folic acid/multivitamin supplementation based on small numbers of cases (Czeizel 1996). However, in a Hungarian case–control study, use of dihydrofolate reductase inhibitors (folic acid antagonists) in pregnancy was associated with a (nonsignificant) 20% excess risk of hypospadias (Czeizel et al. 2001).

Although we cannot exclude recall bias in our study, we believe it is unlikely to explain our findings, given that folate has not previously been associated with hypospadias, and, to explain the association reported here, the size of any such bias would need to be large. In the United Kingdom, 400 μg folate supplementation is recommended in the first trimester of pregnancy for the prevention of neural tube defects (Department of Health 2000). Although we did not collect quantitative data on dietary intake of folate, the National Diet and Nutrition Survey estimated that mean intake of folate from foods ranged from 229 μg/day for women at 19–24 years of age to 255 μg/day at 35–49 years of age (National Diet and Nutrition Survey 2003). Thus, folate supplementation is likely to have more than doubled the daily folate intake. Like the neural tube, the urethra is a midline structure. Biochemical, genetic, and epidemiologic observations suggest that folic acid may prevent neural tube defects by stimulating cellular methylation reactions, although this methylation hypothesis requires further exploration (Blom et al. 2006). Folic acid may also protect against other congenital anomalies such as orofacial clefts, cardiac, and urinary tract defects (Hernandez-Diaz et al. 2000). In addition, recall bias for hair spray is unlikely, because only one previous study reported a possible association (Vrijheid et al. 2003). Recall bias is not an issue for phthalate exposure, because it was assessed by job exposure matrix. However, some exposure misclassification is possible with the job exposure matrix because of uncertainty in expert assessment (Van Tongeren et al. 2002).

Of other potential risk factors for hypospadias, some studies have suggested associations with occupational exposures of the father, including vehicle mechanics and exposure to solvents (Irgens 2000; Pierik et al. 2004). In our study we did not collect information on occupation of the father. Family history (Angerpointner 1984; Brouwers et al. 2007; Hernandez-Diaz et al. 2000; Kallen et al. 1986; Neto et al. 1981) and low birth weight (Angerpointner 1984; Kallen et al. 1986; Neto et al. 1981) have also been reported. Although possible associations with maternal smoking are inconsistent (Angerpointner 1984; Brouwers et al. 2007; Carmichael et al. 2005; Kallen 1997), recent reports suggest that if the father smoked, there was a higher risk of a boy being born with hypospadias than if the mother smoked (Pierik et al. 2004), implying that environmental tobacco smoke (ETS) may play a part. In unadjusted analysis we found a borderline significant increased risk associated with ETS.

Major strengths of our study include the large sample size, wide population-based coverage, and extensive interviewer-based questionnaire. Most previous studies of hypospadias have relied on routinely collected registry data with limited information on potential risk factors and confounders, and varying levels of quality control and completeness (Aho et al. 2003; Irgens 2000; Kallen 1988; Kallen et al. 1986; Kristensen et al. 1997; Vrijheid et al. 2003). We ascertained slightly more cases than the hospital registries and many more cases than the national congenital anomalies system (Nelson et al. 2007). Our study also has limitations. One potential weakness is the low proportion of control women who replied to our invitation to participate. Because of constraints imposed by the ethics committee, we were not able to contact the women in the control group directly after they were randomly selected from the birth registry. Instead, we had to follow a convoluted procedure requiring both health authorities and the mothers’ GPs to forward our invitation pack, with the result that an estimated 72% of nonresponders never received the participant invitations. Although the controls appeared to be of slightly higher social class than the cases, adjusting for socioeconomic status (i.e., income or education) made no difference to our findings.

Furthermore, we investigated more formally the potential for selection bias by socioeconomic status (SES) in selection of cases and controls using a weighting procedure analogous to poststratification for adjustment of item nonresponse in the survey literature (Park et al. 2004). In brief, this involves reweighting the estimates of probabilities of exposure conditional on case/control status according to the distribution of SES in the target (i.e., unbiased population). The idea is that if, for example, individuals with low SES are underrepresented in the study, these estimates can be up-weighted using the SES distribution of the target population. We found no evidence of selection bias mediated by SES in the study (Geneletti et al. 2008).

A particular difficulty affecting research on hypospadias is the wide variation in case definition, from mild displacement of the urethral orifice to severe anomalies requiring major corrective surgery (Baskin et al. 2001). We relied on clinical judgment of surgeons who operate on cases, and thus excluded milder cases who were not referred for surgical correction. We also excluded cases where hypospadias was part of a wider syndrome. Thus our study is likely to have included a greater proportion of more severe, isolated cases than some previous studies.

In conclusion, this is the first study to report increased risks of hypospadias associated with exposure to phthalates and hair sprays and a protective effect of folate supplementation. Previous association of vegetarianism with risk of hypospadias was not confirmed. Measurements of exposure to phthalates and/or biomonitoring may help to understand possible pathways of exposure and toxicology and provide quantitative estimates. Our findings with respect to folate use may have important implications for public health and prevention.

## Figures and Tables

**Table 1:** Potential risk factors and confounders for hypospadias, unadjusted ORs, and 95% CIs.

Characteristic	Cases No. (%)	Controls No. (%)	OR (95% CI)	*p*-Value
Maternal age (years)				0.080
15–24	61 (13.1)	48 (9.8)	1.56 (1.02–2.41)	
25–29	127 (27.2)	106 (21.6)	1.47 (1.06–2.05)	
30–34^a^	165 (35.3)	203 (41.4)	1.00	
35–39	95 (20.3)	109 (22.2)	1.07 (0.76–1.51)	
40–50	19 (4.1)	24 (4.9)	0.97 (0.51–1.84)	
Ethnicity				0.520
Nonwhite^a^	46 (9.8)	42 (8.6)	1.00	
White	425 (90.2)	448 (91.4)	0.87 (0.56–1.34)	
Income				0.001
< £10,000	56 (12.4)	29 (6.1)	2.74 (1.55–4.92)	
£10,000 to < £20,000	130 (28.8)	110 (23.0)	1.68 (1.08–2.62)	
£20,000 to < £30,000	127 (28.2)	131 (27.3)	1.38 (0.89–2.14)	
£30,000 to £50,000	88 (19.5)	138 (28.8)	0.91 (0.58–1.42)	
> £50,000^a^	50 (11.1)	71 (14.8)	1.00	
Gestational age				0.010
Preterm (< 37 weeks)	54 (12.7)	34 (7.5)	1.79 (1.15–2.83)	
Term (≥ 37 weeks)^a^	371 (87.3)	418 (92.5)	1.00	
Birth weight				0.010
Low (< 2,500 g)	57 (13.8)	38 (8.5)	1.71 (1.11–2.66)	
Normal (≥ 2,500 g)^a^	357 (86.2)	407 (91.5)	1.00	
Vegetarian/vegan				0.360
No^a^	390 (83.5)	394 (81.2)	1.00	
Yes	77 (16.5)	91 (18.8)	0.85 (0.61–1.19)	
Maternal smoking^b^				0.020
No^a^	355 (75.5)	397 (81.7)	1.00	
Yes	113 (24.0)	88 (18.1)	1.44 (1.05–1.97)	
Environmental tobacco smoke at home (0–12 weeks of pregnancy)				0.050
No^a^	344 (74.6)	383 (80.5)	1.00	
Yes	114 (24.7)	93 (19.5)	1.36 (1.00–1.86)	
Folate first 3 months during pregnancy^c^				0.010
No^a^	103 (21.9)	77 (15.8)	1.00	
Yes	348 (74.0)	404 (82.8)	0.64 (0.46–0.89)	

a Reference group.

b Two cases and one control stated “don’t know” in answer to the question.

c Nineteen cases and seven controls stated “don’t know” in answer to the question.

**Table 2:** Exposure prevalence, unadjusted ORs, and 95% CIs for self-reported maternal occupational exposure among employed women (for substances with an exposure prevalence > 5%) and unemployed women.

Exposure	Cases No. (%)	Controls No. (%)	OR (95% CI)	*p*-Value
Hair spray				0.004
Not exposed^a^	294 (63.0)	324 (67.8)	1.00	
Exposed^b^	50 (10.7)	24 (5.0)	2.30 (1.38–3.89)	
Unemployed	123 (26.3)	130 (27.2)	1.04 (0.78–1.40)	
Cleaning agents				0.480
Not exposed^a^	233 (49.9)	250 (52.30)	1.00	
Exposed^b^	111 (23.8)	98 (20.5)	1.22 (0.88–1.68)	
Unemployed	123 (26.3)	130 (27.2)	1.02 (0.75–1.38)	
Printing ink				0.810
Not exposed^a^	282 (60.4)	291 (60.9)	1.00	
Exposed^b^	62 (13.3)	57 (11.9)	1.12 (0.76–1.67)	
Unemployed	123 (26.3)	130 (27.2)	0.98 (0.73–1.31)	
Glue				0.290
Not exposed^a^	313 (67.0)	304 (63.6)	1.00	
Exposed^b^	31 (6.6)	44 (9.2)	0.68 (0.42–1.11)	
Unemployed	123 (26.3)	130 (27.2)	0.92 (0.69–1.23)	
Exhaust fumes				0.950
Not exposed^a^	295 (63.2)	299 (62.6)	1.00	
Exposed^b^	49 (10.5)	49 (10.3)	1.01 (0.66–1.56)	
Unemployed	123 (26.3)	130 (27.2)	0.96 (0.71–1.29)	
Grain, hay, paper, and textile dust				0.650
Not exposed^a^	313 (67.0)	323 (67.6)	1.00	
Exposed^b^	31 (6.6)	25 (5.2)	1.28 (0.74–2.23)	
Unemployed	123 (26.3)	130 (27.2)	0.98 (0.73–1.31)	
Dust				0.340
Not exposed^a^	273 (58.5)	291 (60.9)	1.00	
Exposed^b^	71 (15.2)	57 (11.9)	1.33 (0.90–1.96)	
Unemployed	123 (26.3)	130 (27.2)	1.01 (0.75–1.36)	

Model estimates based on data for 371 cases and 419 controls.

a Employed women not occupationally exposed.

b Employed women occupationally exposed.

**Table 3:** Multiple regression models^a^ of hypospadias:adjusted ORs and 95% CIs.

Variable	Adjusted OR (95% CI)	*p*-Value
Income		0.003
< £10,000	2.92 (1.49–5.85)	
£10,000 to < £20,000	1.59 (0.97–2.61)	
£20,000 to < £30,000	1.52 (0.94–2.48)	
£30,000 to £50,000	0.99 (0.60–1.64)	
≥ £50,000	1.00	
Birth weight		0.010
Low (< 2,500 g)	1.87 (1.18–2.98)	
Normal (≥ 2,500 g)	1.00	
Maternal smoking		0.270
No	1.00	
Yes	1.22 (0.85–1.76)	
Folate during first 3 months of pregnancy		0.020
No	1.00	
Yes	0.64 (0.44–0.93)	
Maternal occupational exposure to hair spray		0.004
No	1.00	
Yes	2.39 (1.40–4.17)	
Unemployed	0.97 (0.69–1.37)	
Or		
Maternal occupational exposure to phthalates		0.100
No	1.00	
Yes	3.12 (1.04–11.46)	
Unemployed	0.91 (0.65–1.28)	

a Estimates for income, birth weight, maternal smoking, and folate supplementation are for the model that includes self-reported exposure to hair spray (model fit Akaike Information Criterion 1066). Estimates are essentially unchanged with inclusion instead of maternal occupational exposure to phthalates (model fit Akaike Information Criterion 1072).
